# Promoter Hypermethylation and Expression Changes of *BRCA1* Gene in a Cohort of Sporadic Breast Cancer Cases among Pakistani Population

**DOI:** 10.31557/APJCP.2020.21.8.2395

**Published:** 2020-08

**Authors:** Taqdees Arif, Natasha Anwar

**Affiliations:** 1 *MPhil Molecular Pathology and Genomics, Department of Biological Sciences, Forman Christian College, Lahore, Pakistan. *; 2 *Consultant Molecular Pathologist, Agha Khan University Hospital Lahore Stat Lab, Lahore, Pakistan. *

**Keywords:** BRCA1 promoter hypermethylation, sporadic breast cancer, triple negative breast cancer, epigenetics

## Abstract

**Objective:**

The purpose of our study was to determine the frequency of *BRCA1* promoter hypermethylation and its association with expression changes of *BRCA1* and main morphological features in sporadic breast cancer.

**Methods:**

A retrospective review of cases was performed to select those with specific morphological features suggestive of breast cancer. *BRCA1* promoter hypermethylation and changes in protein expression were evaluated in 30 cancerous and 30 non-cancerous tissue samples. A tissue microarray containing samples from normal and tumor tissue was prepared and stained for BRCA1 protein expression using a commercially available monoclonal antibody against BRCA1 (Ab-1) clone MS110 (mAb). DNA was extracted using modified protocol of Qiagen minikit. DNA was modified using a Bisulfite conversion kit and BRCA1 hypermethylation was detected using a methylation specific PCR.

**Results:**

Promoter hypermethylation was negative in 30 non-cancerous samples with retained BRCA1 protein expression. Methylation was positive in 82.6% (n=19/23) of the sporadic cancer samples that had loss of *BRCA1* expression and 50% (n=2/4) of the samples with equivocal protein expression. Methylation was negative in all the sporadic breast cancer samples (n=3/3) with retained protein expression. Chi-square analysis showed significant association of BRCA1 promoter methylation with decreased protein expression (P=0.016) and co-existence of loss of BRCA1 and Her2neu at chromosome 17 (P=0.026) respectively. There was no significant association of *BRCA1* methylation with morphological features excluding necrosis (P=0.035). Promoter hypermethylation was found to be most common (68.75%) among Triple Negative Breast Cancer (TNBC) females less than 45 years old.

**Conclusion:**

Our study suggests that BRCA1 promoter hypermethylation has significant contribution in sporadic breast carcinogenesis. This was our preliminary study in Pakistan. Further studies aimed to determine the in-depth mechanisms of BRCA1 epigenetics in TNBC. BRCAness enriched phenotype in TNBC might be used as a biomarker for the exploitation of therapeutic and clinical implications.

## Introduction

Breast cancer is a complex disorder, which involves the interplay of various genes and pathways. Pakistan has the highest prevalence of breast cancer in Asia excluding Israel as one in every eight women is suffering from breast cancer. The annual Age Standardized Incidence Rate for breast cancer stood at: 43.3/100,000 females at a global level and 450.3/100,000 females in Pakistan (Badar et al., 2015). However, the frequency of breast cancer has increased by more than 20%, and mortality rate has increased by 14% in Pakistan since past few years (Jemal et al., 2011). Breast cancer occurs in hereditary, familial and sporadic forms. Sporadic breast cancer accounts for 70% of all cases (Gretchen et al., 2010; Nkondjock and Ghadirianet, 2004). Triple negative breast cancer (TNBC) does not express human epidermal growth factor receptor 2 (HER2), estrogen receptor (ER) and progesterone receptor (PR) (Chiorean et al., 2013; Gupta, 2013). Out of all breast cancer subtypes, TNBC is the most aggressive and accounts for 15-30% of breast cancer cases (Chiorean et al., 2013). It accounts for 10-20% worldwide, 25-30% in Asian Population and 34.3% (highest) in Pakistan. Punjabi and Sindhi are the most common ethnic group (Ahmed et al., 2017; Roy et al., 2012).

The TNBC subtype occurs in 18–30% of cases in Pakistan and is associated with early metastasis, rapid growth, younger age of onset and a worse prognosis than other subtypes. Triple negative sporadic tumors often show the same clinical outcome and pathological characteristics as *BRCA1* germline mutation carriers. Several studies hypothesized that *BRCA1* inactivation (BRCAness) might also have a significant contribution in sporadic TNBCs. These tumors can have *BRCA1* promoter methylation or another somatic mutation causing a dysfunctioning BRCA pathway (Pop et al., 2018; Sharma, 2016).

BRCAness can be defined as acquired mutation in DNA repair mechanisms in sporadic breast cancer cells (Pop et al., 2018). Up to 30% of triple negative cases have a germline mutation (Greenup et al., 2013). However, a large frequency of tumors share the molecular characteristics of BRCAness (Quigley et al., 2017) and there is a strong relation between the two entities, leading to a worse prognosis (Temian et al ., 2018). Aberrant DNA methylation regulated by abnormal expression of DNA methyl-transferase is the most well-studied epigenetic change, which culminates in altered gene expressions and BRCAness (Kanwal et al., 2015). The first epigenetic study showed DNA hypermethylation of a *BRCA1* promoter in TNBC (Dobrovic et al., 1997) and its frequency varies from 10 to 85% with higher DNA methylation usually found in more invasive breast tumors (Birgisdottir et al., 2006 ; Southey, 2011).

Breast tumors arising from *BRCA1* promoter hypermethylation and germline mutations acquired distinct morphology. The exact pathogenic mechanism of breast cancer progression is not fully understood yet. Morphological predictors of breast cancer including lymph node status, tumor histological grade, hormonal receptor status, abnormal mitosis, necrosis has shown to be associated with only germline mutations (Zannas et al., 2015). However, epigenetics responds to other risk factors that stimulate gene expression. The exact nature of this outcome still remains to be the subject of debate but these risk factors can include various socioeconomic, dietary, lifestyle and environmental factors (Butcher and Rodenhiser, 2007; Zannas and West, 2014). The difference in morphology could also indicate a difference in timing of these changes during the process of tumorigenesis. Conversely, reversibility of epimutations by PARP (Poly (ADP) ribose polymerase) and DNA methyltransferase inhibitors provides the foundation of novel therapeutic strategies (Li et al., 2014; Pop et al., 2014). *BRCA1* deficient cells are highly sensitive to PARP and DNA methyltransferase inhibition in comparison with wild-type *BRCA1* cells, resulting in clinical trials in breast cancer patients to test the efficiency of these drugs (Dobrovic and Simpfendorfer, 1997). Therefore, BRCAness enriched phenotype can be used as a biomarker for the exploitation of therapeutic options and clinical implications (Wong et al., 2011).

*BRCA1* is a major breast cancer causing gene. It is present on chromosome 17q21 and consists of 24 exons and 1863 amino acids. The most extended character of BRCA deficient cell is DNA double strand break and failure in DNA cross-links repair through accurate homologous recombination. This results in genomic instability and hence the development of cancer (Liede et al., 2002). *BRCA1* germline mutations have been found in inherited breast cancer. Four major Pakistani studies reported the mutational spectrum of *BRCA1* gene (Farooq et al., 2011). Estimated ratio of *BRCA1* germline mutation with respect to inherited breast cancer is 9-17% (Sharma, 2016). However, its prevalence in sporadic breast cancer with respect to Pakistani population has not been very well established yet.

A test for BRCAness may provide a foundation for treatment with PARP and DNA methyltransferase inhibitors. *BRCA1* gene silencing associated with promoter hypermethylation also predicts higher sensitivity to platinum-based drugs to the same extent as *BRCA1* germline mutations. Most importantly, promoter hypermethylation of *BRCA1* has been shown to improved overall survival in ovarian cancer patients undergoing chemotherapy with cisplatin (Butcher et al., 2007; Pop et al., 2018). 

In a resource limited setting it would be advantageous to screen for *BRCA1* promoter methylation as it would enable better management of patients, through targeted therapy. Surrogates of methylation such as immunohistochemical staining with *BRCA1* mAb and methylation specific PCR could provide more cost-effective testing methods. To explore the utility of *BRCA1* promoter methylation screening for breast tumors in Pakistan we investigated the frequency of BRCA 1 promoter methylation and its association with expression changes of *BRCA1* and certain morphological features suggestive of breast cancer in a small cohort of patients.

## Materials and Methods

*Methods*

*Ethics Statement and Sample Selection Criteria*

This study was carried out in accordance with the recommendations of Institutional Review Board Guidelines, Forman Christian College University IRB. The protocol was approved by the Forman Christian College University IRB (ERC-42-2018). A retrospective review of cases was performed to select those with specific morphological features suggestive of breast cancer including; tumor size, tumor grade, histological type, stage, necrosis, lymph node status, lymphocytic infiltrate, abnormal mitotic figures, trabecular growth pattern and hormonal receptors (ER, PR, HER2neu) status. A total of 30 sporadic breast cancer and 30 non-cancerous formalin fixed paraffin embedded (FFPE) tissue blocks were obtained from the Histopathology Section, Chughtais Lahore Lab (CLL).

*Immunohistochemical Staining*

A tissue microarray (TMA) containing samples from each normal and cancerous breast tissue was prepared. TMA was stained for *BRCA1* using a monoclonal antibody (Abcam, Pakistan) against *BRCA1* (Ab-1) clone MS110 (mAb). Sections were cut from the TMA block (5 microns) using a microtome and slides were prepared. Slides were incubated at 60^o^C for 30 minutes. Wax was removed with xylene and tissue sections were rehydrated in absolute ethanol (95%) for 3 minutes. After dewaxing, the sections were stained with the antibody. Slides were incubated in Dako antigen retrieval solution for 1 hour and 30 minutes to maintain pH and retrieve the antigen-binding site that may have been altered by fixation. Then, 50 ul of MS110 (100ug/ul, 1:100 dilutions) primary antibody was applied for 30 minutes. Incubation with secondary antibody (carries the labelled enzyme; Horse radish peroxidase) was done for 20 minutes. Chromogen (Dako, Pakistan) was added to visualize the antibody/antigen complex and incubated at room temperature for 1 minute. Slides were washed with distilled water and counterstaining was done with haematoxylin and eosin, which gives brown color for positive results and purple color for negative results. Slides were washed with distilled water and dehydrated with absolute ethanol. After rehydration, slides were mounted with coverslips. Staining slides were interpreted in context with positive and negative controls, using bright field microscopy at 40X.

*DNA Extraction and Bisulfite Conversion*

Genomic DNA was extracted from the FFPE breast tissue samples using modified protocol of Qiagen minikit. Bisulfite conversion of DNA was done using EPiTect Bisulfite conversion kit (Qiagen, Pakistan).

*Methylation Specific PCR*

Primers were selected from research article (Li et al., 2015). The amplified product of methylated product was 75 bp and unmethylated PCR was 86 bp. PCR reagents were purchased from Abcam (Lahore, Pakistan). Two microliters of Bisulfite converted DNA were amplified in a total volume of 10 ul solution containing 25 mM MgCl_2_, 5 U/ul Hot start taq polymerase, 1X PCR buffer, 10mM dNTPs and 50 pmol of each *BRCA1* primer. The mixture was subjected to methylation specific touchdown PCR profile (Step 1: Initial denaturation at 95°C for 5 minutes followed by 5 cycles of 94°C for 30s, 62°C for 1 min, and 72°C for 30s. Step 2: 5 cycles of 94°C for 30s, 61°C for 1 min and 72°C for 30s. Step 3: 5 cycles of 94°C for 30s, 59°C for 1 min and 72°C for 30s. Step 4: Initial denaturation at 95°C for 10 minutes followed by 35 cycles of 94°C for 15s, 57°C for 30s, 72°C for 30s and final extension of 72°C for 10 minutes). DNA from normal FFPE breast samples was used as a negative control. PCR products were electrophoresed on 2.5% agarose gels and visualized under UV illumination. The presence of methylated product indicated the hypermethylation of *BRCA1* gene. 

*Statistical Analysis*

Pearson chi square test was used to evaluate the association of *BRCA1* promoter methylation status with morphological features and *BRCA1* protein expression. All statistical calculations were done using SPSS software. A P value <0.05 was considered significant.

## Results

*Morphological Features*

Each tumor sample was categorized into different subgroups according to each morphological feature. All the morphological features were not reported in non-cancerous breast tissue samples. The morphological characteristics of the 30 sporadic breast cancer patients are summarized in Table 1. 

*Immunohistochemical Staining*

**Figure 1:**
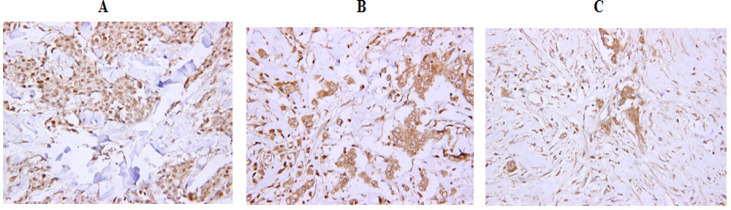
Representative *BRCA1* IHC Stains. High power (40X) images. A: Retained *BRCA1* IHC stain (>10% of tumor nuclei staining), B: Equivocal *BRCA1* IHC stain (5%–10% of tumor cell nuclei staining) and C: Loss of *BRCA1 *(<5% of tumor nuclei staining).

Each IHC stain was reviewed independently by two pathologists and scored as retained, equivocal or absent, equivocal as follows; Retained: >10% of tumor cell nuclei staining similar to the negative internal control, Equivocal: 5%–10% of tumor cell nuclei staining, Loss: <5% of tumor nuclei staining similar to the positive internal control. Loss and equivocal samples were considered tumor and retained samples were considered normal. Figure 1 shows representative IHC stains of *BRCA1*. *BRCA1* expression was retained in 30 non-cancerous breast tissue samples. *BRCA1* expression was loss in 23 sporadic breast cancer patients, equivocal in 4 patients and retained in 3 breast cancer patients. 

*Methylation Specific PCR*

**Figure 2:**
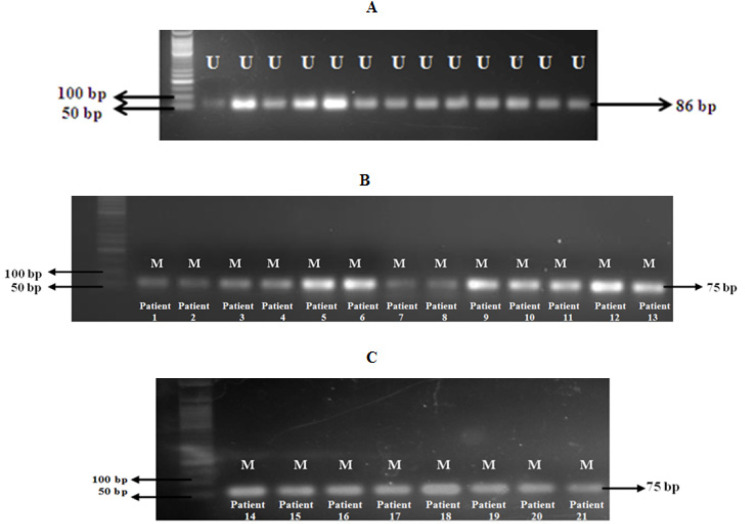
Touchdown MS-PCR Results. (A) Representative image of presence of unmethylated PCR product in cancerous and non-cancerous tissues. (B) and (C) Presence of *BRCA1* promoter hypermethylation in sporadic breast cancer patients (with equivocal and loss of *BRCA1* protein expression)

*BRCA1* promoter hypermethylation was detected using Methylation-Specific PCR. Promoter hypermethylation was negative in non-cancerous breast tissue samples with retained *BRCA1* protein expression. Figure 2 shows the promoter methylation in sporadic breast cancer patients. Methylation was positive in 82.6% (n=19/23) of the sporadic cancer samples that had loss of *BRCA1* expression and 50% (n=2/4) of the samples with equivocal protein expression. Methylation was negative in all the sporadic breast cancer samples (n=3/3) with retained protein expression. Consistent with the *BRCA1* promoter methylation, the protein expression levels of *BRCA1* were reduced in sporadic breast tumors with methylated genes.

*Correlation of BRCA1 Methylation with BRCA1 Protein Expression and Tumor Morphological Features*

Table 1 summarizes the correlation of *BRCA1* promoter methylation with tumor main morphological features and *BRCA1* protein expression. Chi-square analysis revealed no statistically significant association of *BRCA1* promoter methylation with age, tumor size, hormonal receptor status, lymph node status, nuclear grade, histological grade and histological type as P value is greater than 0.05. There was a statistically significant association of *BRCA1* promoter methylation with necrosis (P=0.035), *BRCA1* protein expression (P=0.016) and co-existence of loss of *BRCA1* and Her2neu at chromosome 17 (P=0.026). Promoter methylation was most frequent (68.75%) among TNBC (Triple Negative Breast Cancer) females less than 45 years old. Although there was no association between tumor main pathological characteristics, Promoter hypermethylation was common (80%) in small size tumor (1-3 cm) and tumor stage II (n= 22/30).

**Table 1:** Association of *BRCA1* Hypermethylation with Morphological Features

No.	Features	Overall		Unmethylated		Methylated		P value
		(N=30)		*BRCA1* Gene		*BRCA1 *Gene		
		N	%	N	%	N	%	
1	Age Group							
	<45 Y	16	53.3	5	31.2	11	68.75	
	45-55 Y	10	33.3	4	40	6	60	
	>55Y	4	13.33	0	0	4	100	0.332
2	Tumor Size							
	1-3 cm	10	33.3	2	20	8	80	
	4-6 cm	10	33.3	3	30	7	70	
	7-10 cm	8	26.6	2	25	6	75	0.155
	> 10 cm	2	66.6	2	100	0	0	
3	Histological Grade							
	I	0	0.0	0	0	0	0	
	II	22	73.3	5	22.7	17	77.3	
	III	8	26.6	4	50	4	50	0.149
4	Lymph Node status							
	+	11	36.6	2	18.18	9	81.82	
	-	19	63.3	7	36.84	12	63.15	0.282
5	Nuclear Grade							
	Bland	0		0		0		
	Intermediate	22	73.3	5	22.7	17	77.2	
	Malignant	8	26.6	4	50	4	50	0.149
6	Pushing Margin							
	Yes	0	0.0	0	0	0	0	
	No	30	100	9	30	21	70	0
7	Necrosis							
	Yes	4	13.3	3	75	1	25	
	No	26	86.6	6	23	20	77	0.035
8	Lymphocytic Infiltrate							
	Yes	0	0.0	0	0	0	0	
	No	30	100	9	30	21	70	0
9	Abnormal Mitotic figures							
	Present	30	100	9	30	21	70	
	Absent	0	0	0	0	0	0	0
10	Invasive ductal carcinoma							
	Yes	30	100.0	9	30	21	70	
	No	0	0.0	0	0	0	0	0
11	Hormonal Receptor status							
	Group (ER+, PR+, Her2-)	8	26.67	4	50	4	50	
	Group 2 (ER+, PR+, HER 2+)	4	13.3	0	0	4	100	
	Group 3 (ER-, PR-, Her2+)	2	6.67	0	0	2	100	
	TNBC (ER-, PR-, Her2-)	16	53.3	5	31.25	11	68.75	0.25
12	Trabecular growth Pattern							
	Yes	30	100.0	9	30	21	70	
	No	0	100.0	0	100	0	100	0
13	BRCA1 protein Expression							
	*BRCA1 -*	23	76.6	4	17.4	19	82.6	
	Equivocal	4	13.3	2	50	2	50	
	*BRCA1 +*	3	10.0	3	100	0	0	0.016
No.	Features	Overall		Unmethylated		Methylated		*P *value
		(N=30)		*BRCA1* Gene		*BRCA1 *Gene		
		N	%	N	%	N	%	
14	Expression of both Her2 & BRCA1							
	Her2+, *BRCA1-*	6	20	0	0	6	100	
	Her2-, *BRCA1+*	2	6.6	2	100	0	0	
	Her2-, *BRCA1*-	22	73.3	7	31.8	15	68.2	0.026
	Her2+, *BRCA1*+	0	0	0	0	0	0	

## Discussion

Epigenetic silencing of *BRCA1* has significant contribution in sporadic breast carcinogenesis. Despite many researches have been undertaken on breast cancer epigenetics in other developed countries, but so far none of these has had much impact on treatment and outcome due to heterogeneity of breast cancer. Furthermore, no study has explored the epigenetic modifications of *BRCA1* sporadic breast cancer in Pakistan yet. Hence it is critical to explore and develop the utility of *BRCA1* epigenetic screening in our resource limited settings.

We found statistically significant association of loss of *BRCA1* protein expression with methylation status (P= 0.016), suggesting the significant role of epigenetic silencing in the *BRCA1* promoter region. Methylation was positive in 82.6% of the sporadic breast cancer patients with the loss of *BRCA1* protein expression. The promoter methylation status is significantly higher than previously reported from other population such as Thailand (24.6%), India (45%), Taiwanese (56%) but present similar highest status with Indonesia (80%), France (89.1%) and Vietnamese (82.1%) (Hsu et al., 2013; Wiencke, 2004; Zhang and Longe, 2015). The differences in the frequency of *BRCA1* methylation among different countries may be accounted due to several variables including; study cohort, methodology and population differences due to exposure to specific lifestyle, environmental and dietary risk factors (Zhang and Yu, 2011). 

Furthermore, the correlation between *BRCA1* hypermethylation and the main pathological features of tumor was analyzed. Morphological predictors of breast cancer including lymph node status, tumor histological grade, hormonal receptor status, abnormal mitosis, necrosis has shown to be associated with germline mutations (Zannas et al ., 2015). However, we did not find any significant association of *BRCA1* promoter hypermethylation with tumor main morphological features excluding necrosis (P=0.035). These results were also similar to that reported in a previous study. This may suggest an alternative pathway of tumorigenesis when the BRCA1 protein is silence through promoter methylation in breast sporadic tumors (Farooq et al., 2011).

In addition, three interesting predictions were revealed which might be associated with poor prognosis, although there was no association between tumor main pathological characteristics including; age, tumor stage, tumor size and *BRCA1* hypermethylation. Firstly, Promoter hypermethylation was frequent (80%) in small size tumor (1-3 cm). Secondly, our study also demonstrated that the promoter methylation of *BRCA1* gene is most common in tumor stage II (n= 22/30) and also relates to stage III (n= 8/30). The reasons underlying the mechanism of *BRCA1* methylation were not elucidated yet but these findings are related to the previous study (Wiencke et al., 2004; Zhang and Long 2015). Moreover, *BRCA1* promoter hypermethylation are associated with early onset, poor histological differentiation and triple negative breast cancer (Truong et al., 2014). In the present study, we found *BRCA1* promoter hypermethylation and loss of protein expression in about 68.75% (16/30) of patients with early stage (less than 45 years) triple negative breast cancer. Our finding recommends that *BRCA1* methylation might also have etiological role in the development of triple negative sporadic breast cancer.

Aberrations of chromosome 17 are important molecular events in human breast carcinogenesis. Several tumor suppressor genes and oncogenes are located at close proximity of chromosome 17 have significant contributions in sporadic cancer. Both polysomy or monosomy of chromosome 17 have been observed in breast cancer (Zhang and Yu, 2011). Our study reported that *BRCA1* promoter hypermethylation was significantly associated (P=0.026) with co-existence of loss of both *BRCA1* and Her2neu which is similar to another study (Li et al., 2015). *BRCA1* and* P53* genes have also been reported to be functionally and physically associated. Co-existence of loss of *BRCA1* and *P53* has been found in triple negative sporadic breast cancer (Greenup et al., 2013; Stefansson et al., 2011). Since, no study has reported the combined role of *BRCA1*, *P53* and *Her2neu *located at chromosome 17 in sporadic breast cancer so far. These observations give an interesting idea that regional methylation of *BRCA1* (*17q 12.21*), *p53* (*17p13*) tumor suppressor genes and Her2neu proto-oncogene (*17q21.1*) present at close proximity on chromosome 17 might have significant contributions in triple negative sporadic breast cancer. It has been postulated that breast cancer with *BRCA1* promoter methylation is more aggressive. The benefits of epigenetic research, especially on gene promoter methylation are linked to the treatment and prevention of breast cancer.

*Conclusion and Future Perspective*

In conclusion, our study found that methylation rates in Pakistani breast cancer women is higher than in the literature of few other countries, possibly due to the advanced cancer stages, environmental factors, our lifestyle, lack of awareness and powerful diagnostic and screening tools. The future challenges should involve in-depth investigations of underlying cellular and molecular mechanisms of triple negative breast cancer epigenetics. The union of environmental, genetic and epigenetic factors are postulated to be playing an important role for sporadic breast cancer risk. Because epigenetics changes are reversible and have potential to diagnose cancer at early stage, this knowledge is critical to our basic understanding of cancer susceptibility and etiology. Understanding of these mechanisms ultimately will also accelerate the development of effective tools to diagnose, prevent and treat all types of human cancer. Furthermore, immunohistochemical staining and methylation specific PCR can be explored as a low-cost screening tool for detection of *BRCA1* dysfunction, especially in resource-constrained settings. In addition, PARP and DNA methyl transferase inhibitors might also treat TNBC with BRCAness that are resistant to conventional therapies such as multidrug resistant. Therefore, DNA methylation analysis is significant for the clinical implications of these therapeutic drugs by allowing rational methods to define molecular endpoints and to optimize treatment schedules and trial design for maximum biological effectiveness so that changes induced by demethylating compounds can be monitored and optimized during treatment.

## References

[B1] Ahmed R, Din HU, Afzal S (2017). Clinicopathological characteristics of triple negative breast cancer. PAFM J.

[B2] Badar F, Mahmood S, Faraz R (2015). Epidemiology of breast cancer at the Shaukat Khanum memorial cancer hospital and research center, Lahore, Pakistan. J Coll Physicians Surg Pak.

[B3] Butcher DT, Rodenhiser DI (2007). Epigenetic inactivation of BRCA1 is associated with aberrant expression of CTCF and DNA methyltransferase (DNMT3B) in some sporadic breast tumours. EJC.

[B4] Birgisdottir V, Stefansson OA, Bodvarsdottir SK (2006). Epigenetic silencing and deletion of the BRCA1 gene in sporadic breast cancer. Breast Cancer Res.

[B5] Chiorean R, Braicu C, Berindan-Neagoe I (2013). Another review on triple negative breast cancer Are we on the right way towards the exit from the labyrinth?. Breast J.

[B6] Dobrovic A, Simpfendorfer D (1997). Methylation of the BRCA1 gene in sporadic breast cancer. Cancer Res.

[B7] Farooq A, Naveed AK, Azeem Z, Ahmad T (2011). Breast and ovarian cancer risk due to prevalence of BRCA1 and BRCA2 variants in Pakistani population: a Pakistani database report. J Oncol.

[B8] Greenup R, Buchanan A, Lorizio W (2013). Prevalence of BRCA mutations among women with triple-negative breast cancer (TNBC) in a genetic counseling cohort. Ann Surg Oncol.

[B9] Gupta S (2013). Triple negative breast cancer: A continuing challenge. Indian J Med Paediatr Oncol.

[B10] Gretchen GL, Burke A, Anderson WF (2010). Epidemiology of triple negative breast cancers. Breast Dis.

[B11] Hsu NC, Huang YF, Yokoyama KK (2013). Methylation of BRCA1 promoter region is associated with unfavorable prognosis in women with early-stage breast cancer. PLoS One.

[B12] Jemal A, Bray F, Center MM (2011). Global cancer statistics. CA Cancer J Clin.

[B14] Li Q, Wei W, Jiang YI, Yang H, Liu J (2015). Promoter methylation and expression changes of BRCA1 in cancerous tissues of patients with sporadic breast cancer. Oncol Lett.

[B15] Li D, Bi FF, Chen NN (2014). A novel crosstalk between BRCA1 and poly (ADP-ribose) polymerase 1 in breast cancer. Cell Cycle.

[B16] Liede A, Malik IA, Aziz Z (2002). Contribution of BRCA1 and BRCA2 mutations to breast and ovarian cancer in Pakistan. Am J Hum Genet.

[B17] Nkondjock A, Ghadirian P (2004). Epidemiology of breast cancer among BRCA mutation carriers: an overview. Cancer Lett.

[B18] Pop LA, Cojocneanu-Petric RM, Pileczki V (2018). Genetic alterations in sporadic triple negative breast cancer. Breast J.

[B19] Quigley D, Alumkal JJ, Wyatt AW (2017). Analysis of circulating cell-free DNA identifies multiclonal heterogeneity of BRCA2 reversion mutations associated with resistance to PARP inhibitors. Cancer Discovery.

[B20] Roy R, Chun J, Powell SN (2012). BRCA1 and BRCA2: important differences with common interests. Nat Rev Cancer.

[B21] Sharma P (2016). Biology and management of patients with triple-negative breast cancer. Oncologist.

[B22] Southey MC, Ramus SJ, Dowty JG (2011). Morphological predictors of BRCA1 germline mutations in young women with breast cancer. Br J Cancer.

[B23] Stefansson OA, Jonasson JG, Olafsdottir K (2011). CpG island hypermethylation of BRCA1 and loss of pRb as co-occurring events in basal/triple-negative breast cancer. Epigenetics.

[B24] Temian DC, Pop LA, Irimie AI, Berindan-Neagoe I (2018). The epigenetics of triple-negative and basal-like breast Cancer: current knowledge. JBC.

[B25] Truong PK, Lao TD, Doan TP, Le TA (2014). BRCA1 promoter hypermethylation signature for early detection of breast cancer in the Vietnamese population. Asian Pac J Cancer Prev.

[B26] Wong EM, Southey MC, Fox SB (2011). Constitutional methylation of the BRCA1 promoter is specifically associated with BRCA1 mutation-associated pathology in early-onset breast cancer. Cancer Prev Res.

[B27] Wiencke JK (2004). Impact of race/ethnicity on molecular pathways in human cancer. Nat Rev Cancer.

[B28] Zannas AS, Arloth J, Carrillo-Roa T (2015). Lifetime stress accelerates epigenetic aging in an urban, African American cohort: relevance of glucocorticoid signaling. Genome Biol.

[B29] Zhang L, Long X (2015). Association of BRCA1 promoter methylation with sporadic breast cancers: Evidence from 40 studies. Sci Rep.

[B30] Zannas AS, West AE (2014). Epigenetics and the regulation of stress vulnerability and resilience. Neuroscience.

[B31] Zhang W, Yu Y (2011). The important molecular markers on chromosome 17 and their clinical impact in breast cancer. Int J Mol Sci.

